# Transcription Factor Driven Gene Regulation in COVID-19 Patients

**DOI:** 10.3390/v15051188

**Published:** 2023-05-18

**Authors:** Daniele Santoni, Nimisha Ghosh, Carlo Derelitto, Indrajit Saha

**Affiliations:** 1 Institute for System Analysis and Computer Science “Antonio Ruberti”, National Research Council of Italy, 00185 Rome, Italy; derelittocarlo@yahoo.it; 2 Faculty of Mathematics, Informatics and Mechanics, University of Warsaw, 02-097 Warsaw, Poland; nimishaghosh@soa.ac.in; 3 Department of Computer Science and Information Technology, Institute of Technical Education and Research, Siksha ‘O’ Anusandhan (Deemed to be University), Bhubaneswar 751030, India; 4 Department of Biological, Geological and Environmental Sciences, Alma Mater Studiorum—University of Bologna, 40138 Bologna, Italy; 5 Department of Computer Science and Engineering, National Institute of Technical Teachers’ Training and Research, Kolkata 700106, India; indrajit@nitttrkol.ac.in

**Keywords:** COVID-19, drug repurposing, RNA-Seq expression, SARS-CoV-2, transcription factors

## Abstract

**Simple Summary:**

The present work aims to shed light on the role that Transcription Factors (TFs) play in the alteration of gene expression and regulation driven by COVID-19 infection. In this regard, 19 human transcription factors were selected, since they are predicted to target and potentially regulate human proteins interacting with Spike glycoprotein of SARS-CoV-2. Thirty-one human genes, predicted as targets of these TFs, were selected since they showed statistically significant differences in their correlation values with respect to TFs between healthy and COVID-19 patients. It can be hypothesised that they are major players in the alteration of the regulation pattern driven by COVID-19. In this light, together with the 19 identified TFs, the 31 human genes be considered as potential targets to counteract COVID-19 infection.

**Abstract:**

SARS-CoV-2 and its many variants have caused a worldwide emergency. Host cells colonised by SARS-CoV-2 present a significantly different gene expression landscape. As expected, this is particularly true for genes that directly interact with virus proteins. Thus, understanding the role that transcription factors can play in driving differential regulation in patients affected by COVID-19 is a focal point to unveil virus infection. In this regard, we have identified 19 transcription factors which are predicted to target human proteins interacting with Spike glycoprotein of SARS-CoV-2. Transcriptomics RNA-Seq data derived from 13 human organs are used to analyse expression correlation between identified transcription factors and related target genes in both COVID-19 patients and healthy individuals. This resulted in the identification of transcription factors showing the most relevant impact in terms of most evident differential correlation between COVID-19 patients and healthy individuals. This analysis has also identified five organs such as the blood, heart, lung, nasopharynx and respiratory tract in which a major effect of differential regulation mediated by transcription factors is observed. These organs are also known to be affected by COVID-19, thereby providing consistency to our analysis. Furthermore, 31 key human genes differentially regulated by the transcription factors in the five organs are identified and the corresponding KEGG pathways and GO enrichment are also reported. Finally, the drugs targeting those 31 genes are also put forth. This in silico study explores the effects of transcription factors on human genes interacting with Spike glycoprotein of SARS-CoV-2 and intends to provide new insights to inhibit the virus infection.

## 1. Introduction

COVID-19, the disease caused by SARS-CoV-2, has been disrupting our lives for more than two years now. Viral infection significantly alters host gene expression, which also changes the complex regulation system driven by Transcription Factors (TF). Studying TFs that have an impact on the regulation of genes involved in SARS-CoV-2 infection, as well as their differential role in COVID-19 patients and healthy individuals, can help us to understand how the virus acts so that we can fight against it.

According to [1,2], CoV infection such as SARS-CoV-1, MERS-CoV and SARS-CoV-2 affect host cell transcription, as well as its translation. Additionally, van Hemert et al. [3] has shown the involvement of host TFs in the replication and transcription activity of MERS-CoV. In this regard, Bari et al. [4] investigated human TFs that can bind with the SARS-CoV-2 sequence. Mosharaf et al. [5] explored key regulatory TFs of differentially expressed hub genes (hub-DEGs) and, based on a higher degree of topological measures, selected FOXC1, GATA2, SRF, FOXL1 and YY1 as the top five key regulatory TFs. Thereafter, they considered 10 hub-proteins corresponding to the hub-DEGs and their regulatory 5 key TFs-proteins as drug target receptors and performed their docking analysis with the SARS-CoV-2 3CL protease-guided top listed 90 FDA-approved drugs. In [6], Sardar et al. have considered an interaction network of 2197 human miRNAs leading to the identification of 51 miRNAs interacting with 77 TFs inducing activation or repression, as well as affecting gene expression of linked genes. Chen et al. [7] identified TFs such as CDX2, HNF4, SMAD4 and GATA that bind to the loci of ACE2 and TMPRSS2 in human intestines, having a very big impact on their gene expressions. In [8], Xu et al. evaluated TF such as KLF2 as a therapeutic target for COVID-19, which induced endothelial dysfunction. According to their study, the expression of KLF2 was reduced in endothelial cells of patients, and thus augmenting KLF2 levels may be therapeutically beneficial. Chapola et al. [9] have identified COVID-19 Master Regulators (MRs) from lung tissues of patients who developed the severe form of the disease. Such MRs include transcription factors such as TAL1, TEAD4, EPAS1, ATOH8, ERG, and ARNTL2.

Motivated by the literature, in this work, we have identified 19 TFs targeting the human proteins interacting with Spike glycoprotein of SARS-CoV-2 [10]. Subsequently, gene expressions of COVID-19 patients and healthy individuals are considered for 13 organs such as the blood, brain, eye, heart, intestine, kidney, liver, lung, pancreas, nasopharynx, respiratory tract, stomach, and uterus. The gene expressions are then used to find the correlations between TFs and the corresponding target genes, providing related *p*-values for each organ considering both the datasets of COVID-19 patients and healthy individuals. This led to the identification of key TFs and the most affected organs such as the blood, heart, lung, nasopharynx and respiratory tract. Thereafter, the key genes differentially regulated by the TFs in the five organs were identified based on ranking. This resulted in the identification of 31 genes in the five organs. Furthermore, the KEGG pathways and GO analysis are reported for the 31 identified genes. The KEGG enriched pathways include Vibrio Cholerae infection, Pathogenic Escheria coli infection, Coronavirus disease, AMPK signaling pathway etc. Finally, drugs targeting the identified 31 genes are reported using Enrichr tool [11,12]. In this regard, Amikacin, Ipratropium Bromide, and Captopril are some of the important drugs targeting the 31 genes in order to inhibit the spread of the virus. To the best of our knowledge, this is the first study to provide a global view, considering 13 different organs, of the role that human TFs can play in the differential regulation of genes linked to COVID infection through direct interaction with viral Spike protein. Furthermore, the use of very strict thresholds on P-value tests guarantees reliable results supported by a solid statistical analysis.

## 2. Materials and Methods

This section elaborates the data preparation followed by the discussion of the pipeline of the proposed work.

### 2.1. Data Preparation

In this work, initially, 317 human proteins interacting with Spike glycoprotein of SARS-CoV-2 are considered based on [10]. Thereafter, the TFs targeting the human genes are identified using the procedure mentioned in Section 2.2.1 (the reader can also refer to [13] for more details). Subsequently, mRNA expression data of the TFs and the target human genes for 784 COVID-19 patients and 425 healthy individuals are downloaded from COVID19db [14]. COVID19db has integrated 95 human transcriptomic datasets across 13 organs. The gene expression values of the 13 organs, including the blood, brain, eye, heart, intestine, kidney, liver, lung, pancreas, nasopharynx, respiratory tract, stomach, and uterus, are considered in this work. Please note that the expression data are log2 normalised. The statistics for the data are presented in Table 1. All the expression data are provided in the Supplementary Materials as excel files.

### 2.2. Pipeline of the Work

**Figure 1:**
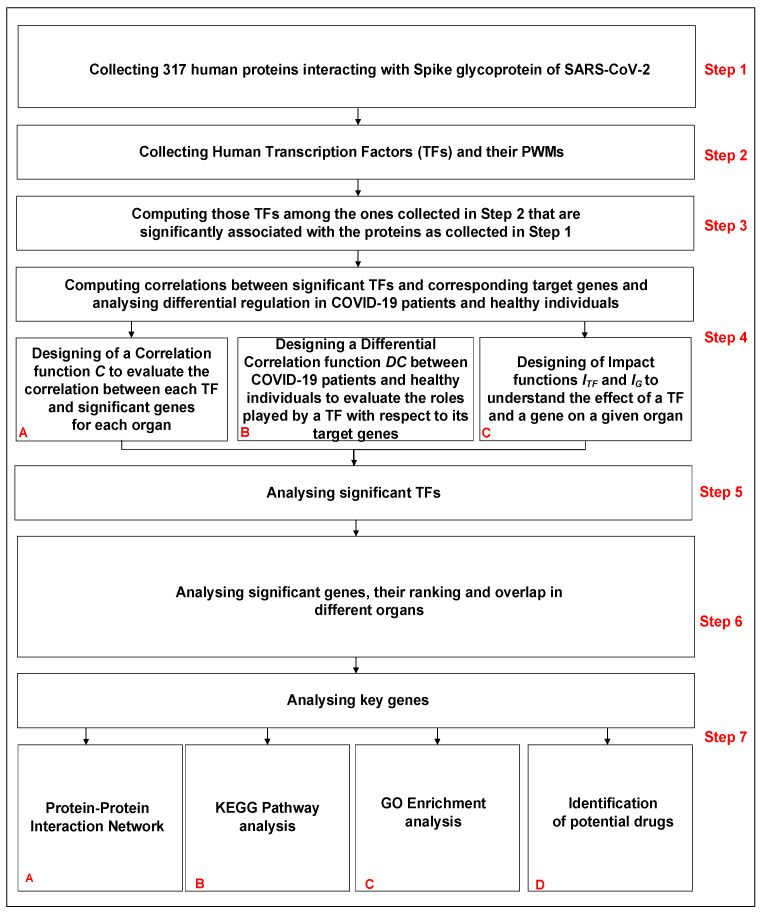
Pipeline of the work.

The pipeline of the work is shown in Figure 1. It consists of seven steps. The first four are related to the collection of data and the methodology, while the last three are related to the analysis and biological interpretation of the obtained results. The steps are as follows:

- Step 1: Collecting human proteins interacting with Spike glycoprotein of SARS-CoV-2.
- Step 2: Collecting human Transcription Factors and their PWMs.
- Step 3: Computing those TFs among the ones collected in Step 2 that are significantly associated with the proteins collected in Step 1.
- Step 4: Computing correlations between significant TFs (Step 3) and corresponding target genes and analysing differential regulation in COVID-19 patients and healthy individuals.
- Step 5: Analysing significant TFs identified in Step 3.
- Step 6: Analysing significant genes, their ranking and overlap in different organs.
- Step 7: Analysing key genes.

Please note that the results obtained from Steps 1–4 are analysed in Steps 5–7.

#### 2.2.1. Identification of Significant TFs

Initially, 317 human proteins which interact with Spike glycoprotein of SARS-CoV-2 are considered (Step 1 of Figure 1). Thereafter, a complete list of 23,459 human genes and 73,432 related transcripts is collected. Promoter sequences (2000 base pairs upstream of the Transcription Start Site are considered according to [15]) of the collected genes/transcripts are retrieved using the package “TxDb Hsapiens UCSC.hg19.KnownGene” version 3.2.2 of R software (https://bioconductor.org/packages/release/data/annotation/html/TxDb.Hsapiens.UCSC.hg19.knownGene.html, accessed on 22 January 2022). A complete list of known and reliable human 626 TFs is selected and the related consensus pattern sequences expressed in terms of Position Weight Matrices (PWMs) are retrieved via the JASPAR database [16] (Step 2 of Figure 1). Transcription Factor binding Sites (TFBSs) associated with each considered human TF are predicted through the matchPWM() function, integrated into the Biostrings R library (https://bioconductor.org/packages/release/bioc/html/Biostrings.html, accessed on 22 January 2022), with a threshold of 0.90. Hypergeometric tests are then performed to assess the association between a given TF and the input set of genes (Step 3 of Figure 1). Such tests are performed for each selected TF in order to evaluate TFBS enrichment in the promoter regions of COVID-19 associated human genes. In other words, the number of genes in the pool set associated with COVID-19 showing at least one TFBS in the promoter region is compared with the number of genes showing at least one TFBS in the set of all human genes, thereby providing the corresponding *p*-value. The obtained *p*-values are then adjusted using Bonferroni’s correction. Significant TFs are finally selected by setting a strict threshold corresponding to an adjusted *p*-value of <$5\times 10^{-2}$. The whole procedure is fully described and the software is available in [13].

#### 2.2.2. Gene—TF Correlations between COVID-19 Patients and Healthy Individuals

mRNA expression data are used to compute Pearson correlation between each selected TF and its target genes (those genes showing at least one predicted TFBS for the given TF) for both COVID-19 patients and healthy individuals for all the 13 considered organs to analyse differential regulation (Step 4 of Figure 1). A significant positive (negative) Pearson correlation between the expression of a given TF and a target gene leads us to hypothesise the potential enhancer (repressive) role that a TF may play in the expression of the considered target gene. We separately evaluate the significance of couples TF—target gene for COVID-19 patients and healthy individuals using the two available datasets. We are mainly interested in those TFs that show different behaviour for COVID-19 and healthy samples. Thus, those couples showing different significant correlation values in the two sample sets are selected. In other words, if a given TF shows a positive correlation with a given gene in a healthy dataset, while it does not in the COVID-19 dataset, it would mean that the regulation process driven by this TF is changed and it is involved in a host altered pathway. In order to perform this kind of analysis, we have firstly designed a function *C* (referred to as Correlation) to evaluate correlation for each organ for the two sample sets separately; we have then designed a second function DC (referred to as Differential Correlation) to evaluate the different roles played by a TF with respect to its target genes in COVID-19 patients and healthy individuals.

The function *C* (Step 4-A of Figure 1) is designed to measure and assess either positive, negative or no correlation and is formally defined as follows:

(1) $$\begin{array}{c} C:TF\times G\times ORG\times [Cov,Heal]\to \{-1,0,+1\}\\ C(tf,g,org,p)\to \{-1,0,+1\} \end{array}$$

where tf∈TF is a transcription factor in the list of significant TFs, g∈G is a gene in the list of COVID-19 associated genes, org∈ORG is one of the 13 considered organs, and *p* is either of the two conditions: COVID-19 or healthy (as reported in the Section of Data Preparation).

- *C(tf,g,org,p)* = −1, if *g* is a target gene of tf (at least one predicted TFBS associated with tf occurs in the promoter region of *g*), the Pearson correlation value between expression data of tf and *g* for the organ org for condition *p* (COVID-19 or healthy) is negative and the associated *p*-value is smaller than $2\times 10^{-6}$.
- *C(tf,g,org,p)* = +1, if *g* is a target gene of tf (at least one predicted TFBS associated with tf occurs in the promoter region of *g*), the Pearson correlation value between expression data of tf and *g* for the organ org for condition *p* (COVID-19 or healthy) is positive and the associated *p*-value is smaller than $2\times 10^{-6}$.
- *C(tf,g,org,p)* = 0, otherwise

**Figure 2:**
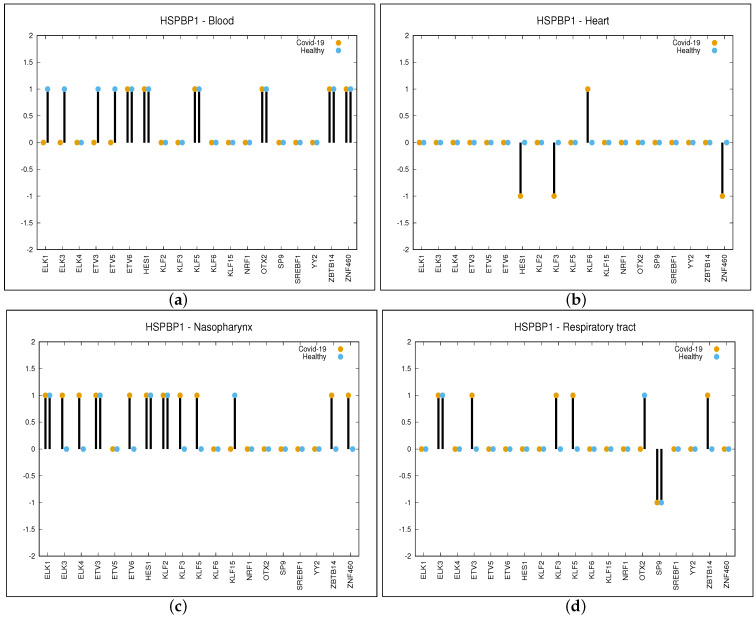
Differential regulation in COVID-19 and Healthy samples of HSPBP1 gene driven by the 19 significant TFs. Values of function *C* are shown: +1 positive correlation, 0 no correlation and −1 negative correlation. Panel (**a**) is related to Blood, panel (**b**) to Heart, panel (**c**) to Nasopharynx and panel (**d**) to Respiratory tract.

The *p*-value of $2\times 10^{-6}$ is due to Bonferroni’s correction on an initial considered *p*-value of $10^{-3}$. This can be attributed to the fact that for each given organ, we have performed a number of tests in the order of thousands (19—the number of TFs—multiplied by the number of target genes resulting in a value of almost 5000). A sample of *C* function values computed for the gene HSPBP1 and four different organs (blood, heart, nasopharynx and respiratory tract) is reported in Figure 2. For the rest of the genes, the figures are provided in Supplementary Materials as FIG_S1. In these figures, only the organs are considered for which the genes are significantly regulated by at least four TFs (this is explained later in the manuscript).

In order to identify those genes that are differentially regulated by significant TFs in COVID-19 and healthy samples for each given organ, we have compared the correlation values of the two conditions: COVID-19 and healthy. We have designed the function DC (Step 4-B of Figure 1) formally defined as follows:

(2) $$\begin{array}{c} DC:TF\times G\times ORG\to \{-2,-1,0,+1,+2\}\\ DC(tf,g,org)=C(tf,g,org,Cov)-C(tf,g,org,Heal) \end{array}$$

where tf, *g* and org are defined as described in Equation (1). For each organ, we have selected as significant the couples {tf,g} whose DC value is either equal to +1 or +2 and the couples {tf,g} whose DC value is either equal to −1 or −2. This can be summarised as follows:

- A strictly positive value of DC for the couple {tf,g} means that the given TF pushes up expression of gene *G* in COVID-19 patients while it does not do so in healthy individuals, or that the given TF pushes down expression of *G* in healthy individuals while it does not do so in COVID-19 patients.
- On the contrary, a strictly negative value of DC for the couple {tf,g} means that the given TF pushes up expression of gene *G* in healthy individuals while it does not do so in COVID-19 patients, or that the given TF pushes down expression of *G* in COVID-19 patients while the inverse is not true.

As can be observed in panel (a) of Figure 2, the DC value for the gene HSPBP1 and organ blood is equal to −1 for the TFs such as ELK1, ELK2, ETV3 and ETV4. This is because for the aforementioned TFs, there is a positive correlation in healthy and no correlation in COVID-19 patients, while the correlation is 0 for the other TFs. When considering the organ heart (panel (b) of Figure 2), DC values are as follows: −1 for the TFs such as HES1, KLF3 and ZNF460 (negative correlation in COVID-19 and no correlation in healthy), +1 for KLF6 (positive correlation in COVID-19 and no correlation in healthy) and 0 for the other TFs. In the same way, we have obtained DC values for nasopharynx and respiratory tract, which are as follows: +1 for ELK3, ELK4, ETV6, KLF3, KLF5, ZBTB14 and ZNF460, −1 for KLF15 in the nasopharynx while +1 for ETV3, KLF3, KLF5 and ZBTB14 and −1 for SP9 in the respiratory tract.

The overall impact of a TF on a given organ can be evaluated based on the number of genes that are differentially regulated between COVID-19 patients and healthy individuals by considering the DC function. In this regard, we have defined the function $I_{TF}$ (Step 4-C of Figure 1) to quantify the impact of a given TF on a given organ, as follows:

(3) $$\begin{array}{c} I_{TF}:TF\times ORG\to \left[0,2\right]\\ I_{TF}\left(tf,org\right)=\sum_{g\in G}\frac{Abs(DC(tf,g,org))}{\left|G\right|} \end{array}$$

where tf, *g* and org are defined as shown in Equation (1), Abs(a) is the absolute value of *a* and |G| is the size of set *G* (for the sake of simplicity, |G| is set to 313, since 4 out of the 317 COVID-19-associated genes have no predicted TFBS for any of the significant TFs).

Similarly, we have defined the function $I_{G}$ (Step 4-C of Figure 1) to evaluate the overall impact of a gene belonging to the COVID-19-associated set.

(4) $$\begin{array}{c} I_{G}:G\times ORG\to \left[0,2\right]\\ I_{G}\left(g,org\right)=\sum_{tf\in TF}\frac{Abs(DC(tf,g,org))}{\left|TF\right|} \end{array}$$

As can be observed in panel (a) of Figure 2, the $I_{G}$ value for gene HSPBP1 and organ blood is equal to four (i.e., the sum of the absolute value of DC for ELK1, ELK3, ETV3 and ETV5, while for the other TFs, DC is equal to zero). In panel (b), it can be observed that $I_{G}$ value for gene HSPBP1 is equal to four for the heart, while it is eight for the nasopharynx (panel (c)) and five for the respiratory tract (panel (d)). Table TAB_S1.xls is provided as supplementary material in which 18 different sheets are reported. Here, 13 sheets are related to each organ considered in this study, while the rest of the 5 sheets are related to the five organs we have focused on. They show the same data, but the genes are ordered with respect to the number of TFs that differentially regulate them. This will help the reader to identify the most significant genes for each of the five organs. Each table (sheet) is related to a given organ and reports differential expression values as a number in the range (−2, +2) so that the reader can easily retrieve each significant couple (non-zero value). For each organ and for each TF, the gene differentially regulated by the given TF can be retrieved, as well as the TFs that differentially regulate the given gene. For example, looking at the table of blood organ column O reports differential regulating values for each gene out of the 313 provided by OTX2.

## 3. Results

CoV infection, as well as any virus infection, significantly alters the gene expression of the host cells, highly impacting the process that regulates transcription and translation processes. In this regard, a central role is played by TFs as one of the main characters involved in regulating gene expression. In this study, a set of TFs significantly associated with COVID-19 infection, as well as the target genes of those TFs showing differential expressions in COVID-19 patients and healthy individuals for each of the 13 considered organs, is identified. The first two subsections correspond to Step 5 in Figure 1, while the third subsection refers to Step 6.

### 3.1. Transcription Factors Significantly Associated with COVID-19 Infection

Predicted TFBSs of known human transcription factors in the promoter regions of all human genes are collected and analysed. For each TF, the collection of identified TFBSs in COVID-19-associated genes is compared with the expected values derived from the analysis of all genes (as reported in the Materials and Methods Section). Hypergeometric tests are performed to identify the list of 19 TFs showing a significant adjusted *p*-value (p<0.05), as reported in Table 2. Many of these TFs play significant roles in COVID-19 progression. As reported in [17], ELK1 can target more than one hub susceptibility gene for COVID-19 in lung adenocarcinoma. According to Melms et al. [18], both AT2 and AT1 cells from COVID-19 lungs showed decreased expression of defining markers. ETV5, which is a transcription factor required for AT2 cell identity, was found to be less expressed in COVID-19 AT2 cells. Reduced ETV5 expression is associated with AT1 cell differentiation, indicating that AT2 cells initiated a regeneration program. KLF2 has been evaluated as a therapeutic target for COVID-19-induced endothelial dysfunction [8]. Lung fibroblasts due to COVID-19 have also been associated with KLF2 [19]. As observed in [20], enrichment of the pathways related to cytokine signalling and inflammation activation in COVID-19 patients is related to KLF6. For the sake of completeness, among the 317 genes interacting with the spike proteins there are 3 TFs (ARNT, CREB3 and STAT1); however, none of them were found to be significant as per the adjusted *p*-value.

### 3.2. Transcription Factors Having a Major Impact on Specific Organs

**Figure 3:**
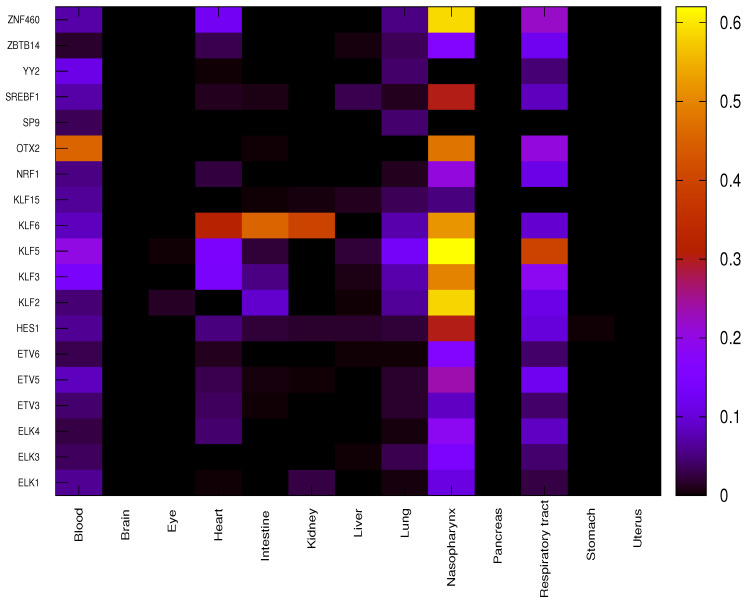
Overall impact of significant TFs in each organ through $I_{TF}$ function. Each cell of the heatmap reports $I_{TF}$ value for a given organ in a color scale.

For each given organ, the correlation between each significant TF and the corresponding target genes is evaluated using function *C* (Equation (1)) for both COVID-19 patients and healthy individuals, providing a score equal to −1 (negative correlation), 0 (no correlation) or +1 (positive correlation). Differential correlation between COVID-19 patients and healthy individuals for each couple {tf,g} is assessed through the function DC (Equation (2)). The overall impact of each TF on a given organ is finally evaluated through the function $I_{TF}$ (Equation (3)) as reported in Figure 3.

It is worth noting that due to the very strict *p*-value threshold, the small number of samples available for some organs makes it very unlikely to find significant correlation. Thus, only a few organs, such as the blood, heart, lung, nasopharynx and respiratory tract, out of the total 13 show significant differential regulation due to specific TFs. OTX2 shows the highest impact in blood, differentially regulating 142 genes out of 313 between COVID-19 and healthy samples. Almost all the concerned genes (139 out of 142) show a DF value equal to −1 leading to hypothesise a suppression of repressive role in COVID-19 patients that was active in healthy individuals. In heart, TF KLF6 shows the highest impact on differential regulation, affecting 100 genes out of 313, with 34 genes showing a value of DF equal to +1 and 66 equal to −1. In lung, the impact values of TFs are much smaller than in other organs, only some TFs belonging to the family KLF (KLF2, KLF3 KLF5 and KLF6) show higher values (20, 23, 42 and 23 out of 313, respectively). It is to be noted that the numbers in () denote the number of genes. Henceforth, this notation will be used throughout.

The nasopharynx shows several very high impact values such as for KLF2 (182 out of 313), KLF3 (155), KLF5 (194), KLF6 (162), OTX2 (149) and ZNF460 (149). Almost all differentially expressed genes show a value of DF equal to +1 so the role of activation of TFs is significantly enhanced in COVID-19 patients with respect to healthy individuals. In respiratory tract, the highest impact value is observed for KLF5 (124 out of 313, with 123 genes showing a DF value equal to +1).

### 3.3. Differentially Regulated Genes in Specific Organs

For each considered organ, genes showing a significant differential regulation due to the role played by TFs are identified through the function $I_{G}$ (Equation (4)). The genes showing a $I_{G}$ value higher than 3 (genes that are differentially regulated in COVID-19 and healthy samples by at least four TFs) are selected: blood (33), heart (13), lung (5), nasopharynx (228) and respiratory tract (50).

**Figure 4:**
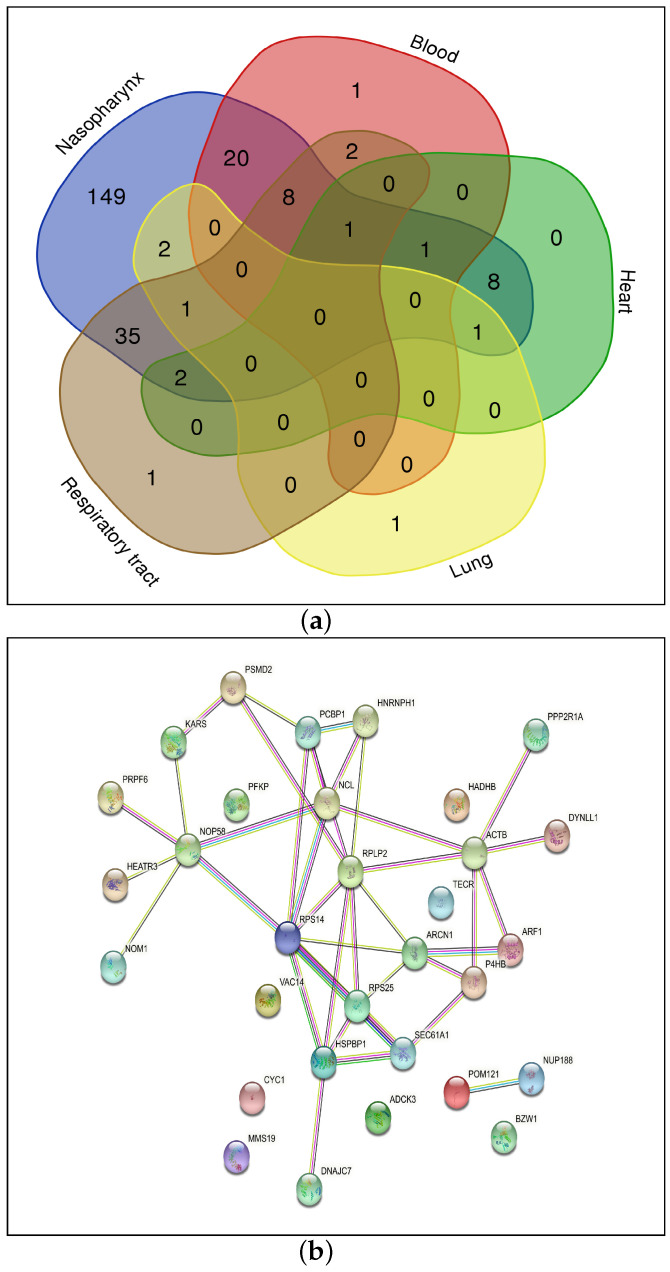
(**a**) Common genes among the 5 organs showing significant differential regulation and (**b**) Protein–Protein Interaction Network.

Figure 4a reports the common genes among five organs: the blood, heart, lung, nasopharynx and respiratory tract. The total number of common genes is 31. Table 3 shows these 31 significant genes by considering the ranking method; for example, a designation of 4* indicates that the gene HSPBP1 is common among four organs such as the blood, heart, nasopharynx and respiratory tract, while 3* indicates that genes such as ARCN1 and PRPF6 are common among organs such as the heart, lung and nasopharynx and so forth.

There are already several vaccines in the market and drugs such as Remdesivir, Paxlovid, molnupiravir and the repurposed rheumatoid arthritis drug baricitinib [21] are also approved by the FDA to combat COVID-19. Thus, drug repurposing can be considered as a good alternative to new drug identification (Step 7-D of Figure 1). However, all the approved drugs target the human proteins directly interacting with Spike protein of SARS-CoV-2. In this work, the focus is on those human proteins that are modulated by the TFs that in turn target the human genes interacting with Spike glycoprotein.

### 3.4. Drugs

**Figure 5:**
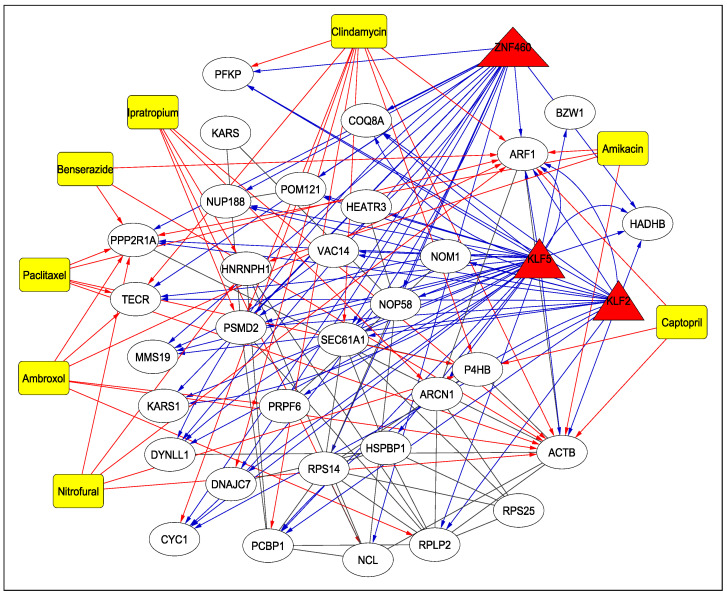
TF–Protein–Drug Interaction Network. The TFs are represented by triangles and are red in colour, the drugs are shown using rectangles and are yellow in colour while the proteins are white in colour and represented by circles. The blue and red arrows show the interaction between TFs and proteins and drugs and proteins, respectively.

Table 4 reports the drugs targeting the 31 human proteins as identified earlier, along with their adjusted *p*-values and DrugBank IDs (https://go.drugbank.com/drugs, accessed on 10 May 2023) (Step 7-C of Figure 1). As can be seen from the table, several drugs such as nitrofural, clindamycin, ipratropium, ambroxol, pacilataxel, benserazisde, amikacin and captopril are used for the treatment of different types of cancer, for Parkinson’s disease and for antibiotic and antibacterial applications in some instances. Among these drugs, Amikacin has been found to be the best aminoglycosides as a potential inhibitor of SARS-CoV-2 [22]. As reported in [23], a 66-year-old man infected with COVID-19 was given Ipratropium Bromide solution, among other drugs, to dilate bronchioles. Another drug, Captopril, is under investigation as a potential drug for COVID-19 [24]. The TF–Protein–Drug interaction network for 3 significant TFs, KLF2, KLF5, ZNF460 and 31 proteins is shown in Figure 5. For example, as can be seen from the figure, KLF2, KLF5 and ZNF460 target ARF1, which is also targeted by drugs such as Nitrofural, Clindamycin, Ambroxol, Paclitaxel, Benserazide, Amikacin and Captopril. All the treatments mentioned in Table 4 have been cited from Drug Bank.

## 4. Discussion

### 4.1. Protein–Protein Interaction Network and KEGG Pathway Analysis

The protein–protein interaction network for the 31 genes as identified and reported in this work is shown in Figure 4b (Step 7-A of Figure 1). Some important pathways corresponding to these genes are shown in Supplementary Figure S1a (Step 7-B of Figure 1). These KEGG pathways are collected from Enrichr tool (https://maayanlab.cloud/Enrichr/, accessed on 10 May 2023). Please note that bar graph rankings are based on *p*-value ranking, q-value ranking, odds ratio ranking and combined score ranking. The bar graph represents high significance based on color and length. The longer and lighter the bar, the more significant the term [25].

As can be seen from the figure, the genes are enriched in pathways which include Vibrio cholerae infection (SEC61A1, ARF1, ACTB), Amyotrophic lateral sclerosis (POM121, NUP188, PSMD2, CYC1, ACTB), Pathogenic Escherichia coli infection (ARF1, NCL, ACTB), Coronavirus disease (RPS14, RPS25, RPLP2), Salmonella infection (ARF1, DYNLL1, ACTB), AMPK signaling pathway (PPP2R1A, PFKP) and Thyroid hormone signalling pathway (ACTB, PFKP). The genes enriched in the pathway for Coronavirus disease are present in the blood, nasopharynx and respiratory tract. It is surprising that some pathways associated with bacterial infection are more significant. At the same time, it is very interesting and this concept deserves more attention and further studies. It can be hypothesised that some common mechanisms which regulate gene expression through TFs are triggered by both COVID-19 and the above-mentioned bacteria. For example, it is known that both COVID-19 and those bacteria are responsible for neurological disorders or alterations in functionality in the mitochondria. Interestingly, two of the genes that are responsible for the enrichment of bacterial infection (*Salmonella*, *Vibrio cholerae* and *Escherichia coli*) pathways are ACTB and ARF1. Both of them are involved in Reactive Oxygen Species (ROS) associated to mitochondria dysfunction [26,27]. This kind of dysfunction was observed in bacterial infections (*Salmonella*, *Escherichia coli* and *Vibrio cholerae*, among others) and also in SARS-CoV2 infection [28,29]. This hypothesis can be the subject of a further study as it is out of the scope of this work.

### 4.2. Gene Ontology (GO) Enrichment Analysis

The significance of the different proteins in biological activities can be shown using GO enrichment analysis (Step 7-C of Figure 1). Similar to KEGG pathways, GO enrichment analysis results for the 31 genes are collected from the Enrichr tool as well. The corresponding results for the biological processes are shown in Supplementary Figure S1b, while the detailed analysis for all the GO pathways (biological, molecular and cellular) are provided on the Supplementary website. Some significant biological pathways are ribosome biogenesis (GO:0042254) (RPS14, NOM1, RPS25, NOP58, HEATR3, RPLP2), gene expression (GO:0010467) (RPS14, RPS25, POM121, NUP188, HNRNPH1, RPLP2, MMS19), SRP-dependent cotranslational protein targeting to membrane (GO:0006614) (RPS14, SEC61A1, RPS25, RPLP2) and cotranslational protein targeting to membrane (GO:0006613) (RPS14, SEC61A1, RPS25, RPLP2).

## 5. Conclusions

For more than two years, COVID-19 has been a major reason for deaths worldwide. To provide a further insight into this deadly disease, in this work, we have identified 19 TFs which target human proteins interacting with SARS-CoV-2 Spike protein. Among the identified TFs, according to the literature, KLF2 has been evaluated as a therapeutic target for COVID-19-induced endothelial dysfunction. Subsequently, mRNA expression data of these 19 TFs and the targeted human genes are considered for both COVID-19-afflicted patients and healthy individuals, focusing on 13 organs. Thereafter, the correlation between the TFs and the genes is carried out to identify the most important TFs, as well as the most affected organs such as the blood, heart, lung, nasopharynx and respiratory tract. Subsequently, 31 common genes are identified whose protein–protein interactions, KEGG pathways as well as GO enrichment are reported. In this work, the focus is on those human proteins that are modulated by the TFs that, in turn, target the human genes interacting with Spike glycoprotein. In this regard, potential repurposable drugs such as Amikacin, Ipratropium Bromide, Captopril, etc., are identified which target the 31 genes. Among these drugs, Captopril is under investigation as a potential drug for COVID-19. We hope that the findings of this work will help the scientific community in the ongoing battle against COVID-19. As a future research direction, this work can be verified by wet-lab experiments as well.

## Figures and Tables

**Table 1:** Statistics for COVID-19 dataset.

GEOID	Organ	Number of Genes	COVID-19	Healthy
GSE163151	Blood	21,952	145	113
GSE164332	Brain	57,996	9	7
GSE164073	Eye	25,222	9	9
GSE162736	Heart	23,194	24	24
GSE159201	Intestine	33,550	12	12
GSE173707	Kidney	24,975	9	9
GSE151803	Liver	22,316	12	9
GSE147507	Lung	20,748	21	29
GSE152075	Nasopharynx	19,744	430	54
GSE165890	Pancreas	22,057	6	6
GSE156063	Respiratory tract	15,811	93	141
GSE153684	Stomach	26,501	9	9
GSE171995	Uterus	19,715	5	3

**Table 2:** Identified significant TFs are reported with corresponding PWM matrix ID, *p*-value and Adjusted *p*-value.

TF	Matrix ID–PWM	*p*-Value	Adjusted *p*-Value
KLF2	MA1515.1	$4.60\times 10^{-12}$	$2.95\times 10^{-9}$
KLF3	MA1516.1	$8.17\times 10^{-10}$	$5.19\times 10^{-7}$
KLF15	MA1513.1	$1.58\times 10^{-9}$	$1.00\times 10^{-6}$
KLF6	MA1517.1	$1.14\times 10^{-8}$	$7.27\times 10^{-6}$
NRF1	MA0506.1	$2.52\times 10^{-7}$	0.00016
SP9	MA1564.1	$4.28\times 10^{-7}$	0.00027
ZNF460	MA1596.1	$9.56\times 10^{-7}$	0.00061
ELK3	MA0759.1	$1.38\times 10^{-6}$	0.00088
HES1	MA1099.2	$5.99\times 10^{-6}$	0.00381
ZBTB14	MA1650.1	$6.58\times 10^{-6}$	0.00418
YY2	MA0748.2	$1.33\times 10^{-5}$	0.00845
ETV3	MA0763.1	$2.03\times 10^{-5}$	0.01290
ELK1	MA0028.2	$2.35\times 10^{-5}$	0.01493
OTX2	MA0712.2	$2.51\times 10^{-5}$	0.01594
ETV5	MA0765.2	$5.00\times 10^{-5}$	0.03179
ETV6	MA0645.1	$5.68\times 10^{-5}$	0.03611
KLF5	MA0599.1	$6.04\times 10^{-5}$	0.03843
SREBF1	MA0829.2	$6.12\times 10^{-5}$	0.03895
ELK4	MA0076.2	$6.44\times 10^{-5}$	0.04098

**Table 3:** Final set of 31 Genes and the related organs.

Designation	Organs	Genes
4*	Blood, Heart, Nasopharynx, Respiratory tract	HSPBP1
3*	Blood, Nasopharynx, Respiratory tract	P4HB
	Blood, Nasopharynx, Respiratory tract	RPS14
	Heart, Lung, Nasopharynx	ARCN1
	Heart, Nasopharynx, Respiratory tract	PRPF6
	Lung, Nasopharynx, Respiratory tract	BZW1
	Blood, Nasopharynx, Respiratory tract	RPS25
	Blood, Nasopharynx, Respiratory tract	NCL
	Blood, Nasopharynx, Respiratory tract	HEATR3
	Blood, Heart, Nasopharynx	HNRNPH1
	Blood, Nasopharynx, Respiratory tract	NOP58
2*	Nasopharynx, Respiratory tract	ARF1
	Heart, Nasopharynx	POM121
	Nasopharynx, Respiratory tract	COQ8A
	Nasopharynx, Respiratory tract	PCBP1
	Nasopharynx, Respiratory tract	DYNLL1
	Blood, Nasopharynx	TECR
	Nasopharynx, Respiratory tract	ACTB
	Nasopharynx, Respiratory tract	MMS19
	Heart, Nasopharynx	PFKP
	Blood, Nasopharynx	HADHB
1*	Nasopharynx	PPP2R1A
	Nasopharynx	RPLP2
	Nasopharynx	NUP188
	Nasopharynx	VAC14
	Nasopharynx	NOM1
	Nasopharynx	CYC1
	Nasopharynx	SEC61A1
	Nasopharynx	PSMD2
	Nasopharynx	DNAJC7
	Nasopharynx	KARS1

**Table 4:** Possible Drugs and their corresponding details.

Human Genes	Drugs	Adjusted *p*-Value	Drug Bank ID	Treatment
ARF1, PPP2R1A, HNRNPH1, P4HB, ACTB	Nitrofural	0.0052	DB00336	Topical antibacterial for the prevention and treatment of bacterial infections of the skin
SEC61A1, ARF1, DNAJC7, PCBP1, PSMD2, TECR, CYC1, P4HB, ACTB, PFKP	Clindamycin	0.0077	DB01190	Antibiotic used to treat serious infections caused by susceptible anaerobic, streptococcal, staphylococcal and pneumococcal bacteria
HNRNPH1, PSMD2, NCL, ACTB, ARCN1	Ipratropium Bromide	0.0101	DB00332	Used in the control of symptoms related to bronchospasm in chronic obstructive pulmonary disease (COPD)
ARF1, PRPF6, PPP2R1A, TECR, RPLP2, ACTB	Ambroxol	0.0130	DB06742	Airway secretion clearance therapy
ARF1, PPP2R1A, TECR, P4HB, ACTB	Paclitaxel	0.0133	DB01229	Treatment of advanced carcinoma of the ovary, and other various cancers including breast and lung cancer
ARF1, PPP2R1A, ACTB	Benserazide	0.0236	DB12783	Treat Parkinson’s disease, Parkinsonism, and restless leg syndrome
ARF1, PPP2R1A, HNRNPH1, ACTB	Amikacin	0.0351	DB00479	An aminoglycoside used to treat infections caused by more resistant strains of Gram-negative bacteria and some Gram-positive bacteria
ARF1, P4HB, ACTB	Captopril	0.0420	DB01197	An ACE inhibitor used for the management of essential or renovascular hypertension, congestive heart failure, left ventricular dysfunction following myocardial infarction and nephropathy

## Data Availability

The supplementary materials for this work are available at http://www.nitttrkol.ac.in/indrajit/projects/COVID-TF/.

## Supplementary Materials

The following supporting information can be downloaded at: https://www.mdpi.com/article/10.3390/v15051188/s1, Figure S1: FIG_S1 (Differential Correlation of Genes with respect to the 19 TFs); Table S1: TAB_S1 (Significant Genes for 5 organs).

## Abbreviations

The following abbreviations are used in this manuscript:

TF	Transcription Factors
TFBS	Transcription Factor Binding Site
C	Correlation
DC	Differential Correlation
PWM	Position Weight Matrix
KEGG	Kyoto Encyclopedia of Genes and Genomes
GO	Gene Ontology
