# Supplementary material for: Transcription Factor Driven Gene Regulation in COVID-19 Patients
Source: Viruses. 2023 May 18;15(5):1188. doi: 10.3390/v15051188 (PMC10223727; doi:10.3390/v15051188)
Supplement: Supplementary file 1 [file viruses-15-01188-s001.zip › Supplementary.pdf]

Supplementary for "Transcription Factor Driven Gene Regulation in COVID-19 Patients"

Daniele Santoni [1,*,†], Nimisha Ghosh [2,3,†], Carlo Derelitto [1,4], and Indrajit Saha [5]

1 Institute for System Analysis and Computer Science "Antonio Ruberti", National Research Council of Italy, 00185 Rome, Italy
2 Faculty of Mathematics, Informatics and Mechanics, University of Warsaw, 02-097 Warsaw, Poland
3 Department of Computer Science and Information Technology, Institute of Technical Education and Research, Siksha 'O' Anusandhan (Deemed to be University), Bhubaneswar 751030, India
4 Department of Biological, Geological and Environmental Sciences, Alma Mater Studiorum—University of Bologna, 40138 Bologna, Italy
5 Department of Computer Science and Engineering, National Institute of Technical Teachers' Training and Research, Kolkata 700106, India
***** Correspondence: daniele.santoni@iasi.cnr.it
† These authors contributed equally to this work.

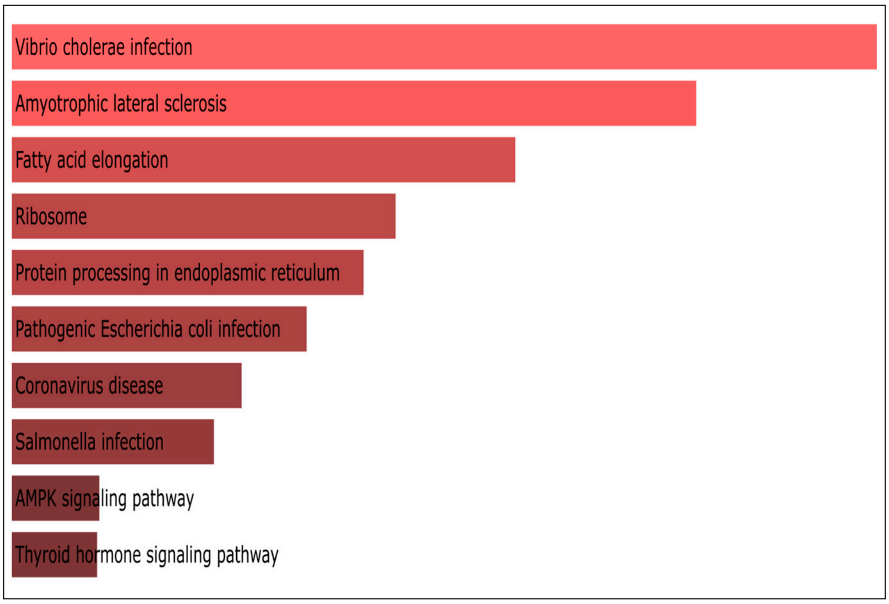

(a)

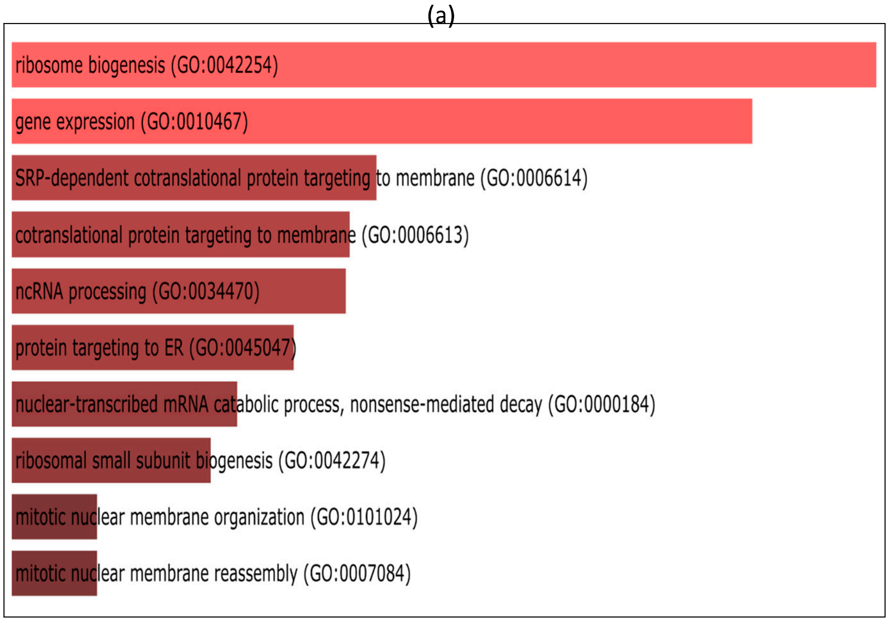

(b)

Figure S1: Significant KEGG pathways and (b) Significant GO Enrichment Analysis for the 31 identified genes
